# Modeling and Estimation of CO_2_ Emissions in China Based on Artificial Intelligence

**DOI:** 10.1155/2022/6822467

**Published:** 2022-07-07

**Authors:** Pan Wang, Yangyang Zhong, Zhenan Yao

**Affiliations:** ^1^State Key Laboratory of Nuclear Resources and Environment, East China University of Technology, Nanchang, Jiangxi 330013, China; ^2^School of Information Engineering, East China University of Technology, Nanchang, Jiangxi 330013, China; ^3^Jiangxi Engineering Laboratory on Radioactive Geoscience and Big Data Technology, East China University of Technology, Nanchang 330013, Jiangxi, China; ^4^State Key Laboratory of Palaeobiology and Stratigraphy (Nanjing Institute of Geology and Palaeontology, CAS), Nanjing, Jiangsu 210008, China; ^5^Engineering Research Center for Seismic Disaster Prevention and Engineering Geological Disaster Detection of Jiangxi Province (East China University of Technology), Nanchang, Jiangxi 330013, China

## Abstract

Since China's reform and opening up, the social economy has achieved rapid development, followed by a sharp increase in carbon dioxide (CO_2_) emissions. Therefore, at the 75th United Nations General Assembly, China proposed to achieve carbon peaking by 2030 and carbon neutrality by 2060. The research work on advance forecasting of CO_2_ emissions is essential to achieve the above-mentioned carbon peaking and carbon neutrality goals in China. In order to achieve accurate prediction of CO_2_ emissions, this study establishes a hybrid intelligent algorithm model suitable for CO_2_ emissions prediction based on China's CO_2_ emissions and related socioeconomic indicator data from 1971 to 2017. The hyperparameters of Least Squares Support Vector Regression (LSSVR) are optimized by the Adaptive Artificial Bee Colony (AABC) algorithm to build a high-performance hybrid intelligence model. The research results show that the hybrid intelligent algorithm model designed in this paper has stronger robustness and accuracy with relative error almost within ±5% in the advance prediction of CO_2_ emissions. The modeling scheme proposed in this study can not only provide strong support for the Chinese government and industry departments to formulate policies related to the carbon peaking and carbon neutrality goals, but also can be extended to the research of other socioeconomic-related issues.

## 1. Introduction

Global warming has become a fact generally accepted by the international community. Climate warming has seriously affected the living environment and social development of humankind. Although the cyclical changes in the natural environment itself affect global climate change, more and more studies have shown that human activities have accelerated the global warming process largely. Since the industrial revolution, with the development of social economy and the increasing intensity of human activities, the concentration of carbon dioxide (CO_2_) in the atmosphere has risen sharply [1]. Studies have shown that the main cause of global warming in the past 50 years is the massive emission of greenhouse gases. The main greenhouse gas is CO_2_, and its emissions are inextricably linked to the extensive use of fossil fuels and the widespread destruction of forests. [2–5]. The fourth assessment report of IPCC (Intergovernmental Panel on Climate Change) pointed out that China's CO_2_ emissions exceeded that of the United States in 2006, ranking first in the world [6]. Moreover, China's CO_2_ emissions have continued to increase in recent years. In 2012, the annual emissions reached 8106.43 million tons. Socioeconomic development is necessary to improve living standards and social welfare. To this end, the government must maintain a stable economic growth rate in the country. Therefore, all countries in the world are facing the dual challenges of CO_2_ emissions and social-economic development. On the one hand, it is necessary to curb the level of CO_2_ emissions caused by the consumption of fossil fuels, and on the other hand, the economic growth rate needs to be maintained [2, 7]. China's economy has grown exponentially since China implemented its reform and opening-up policy. Studies have shown that China's GDP has grown by an average of more than 9% per year in the past four decades [8–10]. However, this rapid GDP growth has been achieved through a massive increase in energy consumption and the accompanying increase in CO_2_ emissions. In recent years, due to the increasingly strong voice in the international community for the development of a low-carbon economy, the issue of CO_2_ emissions has attracted widespread attention from scholars around the world, and some research results have been achieved. Scholars' research on CO_2_ emissions mainly focuses on two aspects, one is to explore the social and economic factors that affect CO_2_ emissions, and the other is to establish CO_2_ emissions prediction models.

The factors affecting CO_2_ emissions are complex and diverse. In recent years, many experts have done a lot of research work on CO_2_ emissions and published some research results. Zha et al. [11] built an assessment framework for direct and indirect CO_2_ emissions from the tourist sector and used the SBM-Undesirable model to integrate the CO_2_ emission component in the efficiency evaluation framework. Liu et al. [12] examined the dynamic relationship between tourism income, economic growth, energy consumption, and CO_2_ emissions. Mujtaba et al. [13] examined the correlation between economic growth, energy consumption, population, trade openness, and CO_2_ emissions. By using the generalized method of moments (GMM) dynamic panel model, Al-Ayouty et al. [14] found a positive correlation between population and CO_2_ emissions. Aller et al. [15] comprehensively analyzed the factors associated with CO_2_ emissions using Bayesian model. The researchers' study was based on data from different countries and the results varied widely [16], but there were some commonalities. Many researchers have found that economic growth and population growth are two of the most important factors that influence CO_2_ emissions. Economic growth affects CO_2_ emissions mainly through three channels: scale effect, structural effect and technical effect [17, 18]. The scale effect of the economy has a promoting effect on CO_2_ emissions, while the structural effect and technological effect have a curb effect on CO_2_ emissions [19]. In a study based on provincial panel data, Wang et al. [20] found that economic growth was an important driver of CO_2_ emissions growth. Using the generalized index method, Shao et al. [21] found that the scale effect of economy is the main contributor to the increase in CO_2_ emissions. Therefore, an increasing number of scholars are currently working to estimate the link between GDP growth and CO_2_ emissions [8, 22–26]. Guo et al. [27] found a positive correlation between population size and CO_2_ emissions. Ohlan [28] studied the relationship between population density, energy consumption, GDP growth, and CO_2_ emissions and found that population density has a strong positive impact on CO_2_ emissions, which is the main factor affecting CO_2_ emissions. In addition, many other factors can affect CO_2_ emissions [29]. Previous research results show that there are many factors affecting CO_2_ emissions, and the impact mechanism is relatively complex. The relationship between CO_2_ emissions and its influencing factors varies greatly due to regional differences [30].

At present, the research on the impact mechanism of CO_2_ emissions is relatively mature. Recently, many researchers have begun to focus on the modeling and prediction of CO_2_ emissions issue. Sun et al. [31] have established CO_2_ emission prediction models based on BP neural network and least square support vector machine, respectively, and found that the least square support vector machine model has better prediction effect. Budiono et al. [32] used a multiple linear regression model to predict CO_2_ emissions from electricity production in Indonesia and the results show that the model predictions are very close to the actual data. Mardani et al. [33] proposed a neuro-fuzzy model to predict CO_2_ emissions, and the results show that the model can effectively predict CO_2_ emissions. Gao et al. [34] proposed a fractional grey Riccati model (FGRM (1, 1)); by testing 20 data sets from M-competition, it is shown that the proposed model can be used to predict short-term CO_2_ emissions. Zhao and Yang [35] tested the dynamic mode decomposition (DMD) for CO_2_ emission prediction, which proves that DMD model has ideal effect for short-term carbon dioxide prediction. Wen and Yuan [36] have designed a new hybrid prediction model (RF-DPSO-BP) for CO_2_ emission prediction and tested the validity of the model through the panel data test of Chinese commercial sector. It can be seen that machine learning methods have been gradually applied to CO_2_ emission modeling and prediction, and existing studies have also confirmed the feasibility and effectiveness of machine learning for CO_2_ emission prediction. However, few studies at the current stage consider the hyperparameters optimization problem of intelligent algorithms such as machine learning applied to CO_2_ emission modeling and prediction. Therefore, this research will focus on two aspects of China's CO_2_ emissions. On the one hand, based on the data of China's CO_2_ emissions and socioeconomic indicators, it will analyze the correlation between CO_2_ emissions and various socioeconomic indicators. On the other hand, considering that the CO_2_ emission modeling data belong to a small sample training set, this study will use the LSSVR to carry out research work. In order to solve the hyperparameter optimization problem of machine learning modeling, the improved artificial bee colony algorithm is mixed with LSSVR to build a CO_2_ emission prediction model based on the hybrid intelligent algorithm. The purpose of this hybrid intelligence model is to accurately predict future CO_2_ emissions, which can provide reliable theoretical support for Chinese government departments to formulate policies to achieve dual carbon (carbon peaking and carbon neutrality) goals. Finally, this study will give some policy recommendations to the Chinese government.

## 2. Theory and Methodology

### 2.1. Methodology of Least Square Support Vector Regression

The basic idea of LSSVR is to use the known sample data to obtain a best fitting function [37] and then input new sample data according to this function to calculate the corresponding output value. Given a training sample set,

(1) $$\begin{array}{c} T=\left\{\left(x_{1},y_{1}\right),\ldots ,\left(x_{l},y_{l}\right)\right\}\in {\left(R^{n}\times Y\right)}^{l}, \end{array}$$

where *x*_*i*_ ∈ *R*^*n*^,  *y*_*i*_ ∈ *Y*=*R*,  *i*=1,2,…, *l*. The sample input is mapped to a high-dimensional space by nonlinear mapping, and the LSSVR function is constructed as follows:

(2) $$\begin{array}{c} y\left(x\right)=\omega^{Τ}\varphi \left(x\right)+b, \end{array}$$

where *ω* is weight vector and *b* is deviation. The solution of (2) can be transformed into an optimization problem, as in

(3) $$\begin{array}{c} \left\{\begin{array}{cc} \underset{\omega ,b,\xi }{\min}, & R=\frac{1}{2}{\left\|\omega \right\|}^{2}+\frac{c}{2}\sum_{i=1}^{l}\xi^{2},\\ \text{s.t,} & y_{i}=\omega^{Τ}\varphi \left(x_{i}\right)+b+\xi_{i}, \end{array}\right. \end{array}$$

where *c* is penalty parameter and *ξ*_*i*_ ≥ 0 is relaxation factor. The function model of LSSVR can be solved by using Lagrange function and KKT optimization condition:

(4) $$\begin{array}{c} y\left(x\right)=\sum_{i=1}^{l}a_{i}K\left(x,x_{i}\right)+b, \end{array}$$

where *a*_*i*_ is Lagrange multiplier and *K*(*x*, *x*_*i*_) is a kernel function. In this study, the Gauss radial basis function with strong fitting ability is used as the kernel function of LSSVR, as in

(5) $$\begin{array}{c} K\left(x,x_{i}\right)=\exp\left(-\frac{{\left\|x-x_{i}\right\|}_{2}^{2}}{2\sigma^{2}}\right). \end{array}$$

### 2.2. Methodology of Adaptive Artificial Bee Colony (AABC) Algorithm

#### 2.2.1. Artificial Bee Colony Algorithm

As a new biomimetic optimization algorithm, artificial bee colony (ABC) has been widely used in recent years. In the ABC algorithm, a honey source is used to represent a function solution, and the quality of the honey source reflects the merits of the solution.

In the ABC algorithm, the bee colony is composed of a lead bee, a follower bee, and a scout bee, and the problem is solved by information exchange, conversion, and mutual cooperation between the three types of bee colonies. When solving the optimization problem, the spatial point is regarded as the location of the honey source, each point represents a possible solution, and *N* represents the number of honey sources. The quality of honey source *x*_*i*_ (*i*=1,2,3,…, *N*) corresponds to the fitness value of the solution. Suppose the dimension of the problem to be solved is *D*, at the *t*th iteration, the position of the honey source is *X*_*i*_^*t*^=(*x*_*i*1_^*t*^*x*_*i*2_^*t*^ … *x*_*iD*_^*t*^)^*T*^, where *t* is the current number of iterations, and the maximum number of iterations is *T*. *x*_*id*_ ∈ (*L*_*d*_, *U*_*d*_), and *M*_*d*_ and *N*_*d*_ respectively represent the two boundaries of the search space, *d*=1,2,3,…, *D*. The ABC algorithm is initialized, and the initial position of the honey source is randomly generated in the search space according to formula (6). Then, the lead bee searches for a new source of honey is according to formula (7).

(6) $$\begin{array}{c} x_{id}=M_{d}+\text{rand}\left(0,1\right)\left(N_{d}-M_{d}\right), \end{array}$$

(7) $$\begin{array}{c} v_{id}=x_{id}+\eta \left(x_{id}-x_{jd}\right), \end{array}$$

where *d* represents a dimension in the solution space; *j* ∈ {1,2,3,…, *N*},  *j* ≠ *i*; *η* is a random number between [−1, 1], representing the magnitude of the disturbance.

The fitness of the two honey sources is then evaluated and the retention of *x*_*i*_ or *v*_*i*_ is determined according to the greedy algorithm. The fitness value of the solution is calculated according to (8), where the function value of the solution is represented. The probability that the honey source found by the lead bee is followed is calculated according to (9).

(8) $$\begin{array}{c} {\text{fit}}_{i}=\left\{\begin{array}{c} \frac{1}{\left(1+f_{i}\right)},\;f_{i}\geq 0,\\ 1+abs\left(f_{i}\right),\;f_{i}<0, \end{array}\right. \end{array}$$

(9) $$\begin{array}{c} P_{i}=\frac{{\text{fit}}_{i}}{\sum_{i=1}^{N}{\text{fit}}_{i}}. \end{array}$$

The follower bee uses roulette to choose to lead bee; that is, a uniformly distributed random number *r* is generated in [0,1]. When *P*_*i*_ > *r*, the follower bee will generate a new honey source around the honey source and use the greedy algorithm to determine the retained honey source. If a better honey source is not found after *t* iterations reach the threshold limit, the honey source *x*_*i*_^*t*^ is discarded. At this time, the lead bee becomes a scout bee, a new honey source *x*_*i*_^*t*+1^ is randomly generated in the search space instead of *x*_*i*_^*t*^, and the calculation formula of *x*_*i*_^*t*^ is 10. On the contrary, the honey source *x*_*i*_^*t*^ is retained. At this time, *t*=*t*+1, and it is judged whether the algorithm reaches the termination condition and the optimal solution is output.

(10) $$\begin{array}{c} x_{i}^{t+1}=\left\{\begin{array}{c} L_{d}+\text{rand}\left(0,1\right)\left(U_{d}-L_{d}\right),\;t\geq \text{limit,}\\ x_{i}^{t},\;t<\text{limit}. \end{array}\right. \end{array}$$

#### 2.2.2. Improved Artificial Bee Colony Algorithm

Two key problems are involved in the ABC algorithm. One is the selection of the search step size. The search range and search accuracy of the population are controlled by the step size *η*, but the value of *η* is completely random. When the value of *η* is too large, it is easy to jump out of the true global optimal solution; when the value of *η* is too small, it is easy to converge earlier and obtain a local optimal solution. According to the analysis of bee feeding behavior, there should be a large search step in the early stage of the search, so as to quickly gather around the global optimal solution and improve the convergence speed. In the later stage of the search, the optimal solution obtained by the algorithm search is close to the real optimal solution. At this time, the search step size should be gradually reduced to achieve a detailed search around the optimal solution. For this reason, this study designs a self-adaption. The step size of the search strategy is adapted to the decreasing operator, and the calculation formula is

(11) $$\begin{array}{c} \left\{\begin{array}{c} v_{id}=x_{id}+\eta \left(x_{id}-x_{jd}\right),\\ \eta =\eta_{0}\left(1-\frac{1}{2}\log_{T}^{t}\right), \end{array}\right. \end{array}$$

where *η*_0_ ∈ (0,1);  *t*={1,2,3,…, *T*}.

Another key issue is the selection of honey sources in the ABC algorithm. In the standard ABC algorithm, only the honey source with high fitness is selected, and the honey source with low fitness is quickly eliminated. This will lead to a decline in the diversity of the population, which will make it difficult for the algorithm to achieve global optimality. Aiming at this problem, this study designs a new adaptive probability selection mechanism, which is calculated as

(12) $$\begin{array}{c} \left\{\begin{array}{c} P_{i}=\lambda \frac{{\text{fit}}_{i}}{\sum_{i=1}^{N}{\text{fit}}_{i}}+\left(1-\lambda \right)\frac{1/{\text{fit}}_{i}}{\sum_{i=1}^{N}1/{\text{fit}}_{i}},\\ \lambda =e^{\left(t/T\right)\ln2}-1, \end{array}\right. \end{array}$$

where *λ* ∈ (0,1). In the early stage of the algorithm, *t*/*T*⟶0, *λ*⟶0. At this time, the honey source with poor fitness has the possibility of being selected, thereby improving the ability of the algorithm to obtain the generalization; in the later stages of the algorithm's operation, *t*/*T*⟶1, *λ*⟶1. At this time, only the honey source with high fitness is selected. Thereby, the convergence speed of the algorithm is accelerated, and the convergence precision of the algorithm is also improved.

In summary, this study designs the adaptive step-down operator to update the step size of the search strategy and designs the adaptive probability selection mechanism to solve the honey source selection problem, improves the ABC algorithm, and establishes an AABC algorithm.

## 3. Data Preparation and Analysis

### 3.1. Data Preparation

**Figure 1:**
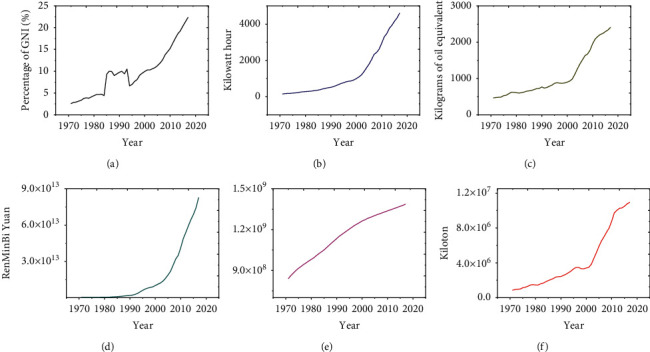
Time-dependent curve of socioeconomic indicators related to CO_2_ emissions. (a) FCC. (b) PC per capita. (c) OC per capita. (d) GDP. (e) Population. (f) CO_2_ emissions.

Database preparation is a very important step for neural network modeling. In this study, the data used were derived from five socioeconomic indicators related to CO_2_ emissions in China from the World Development Indicators database published by the World Bank, including fixed capital consumption (FCC), power consumption (PC), oil consumption (OC), gross domestic product (GDP), population (P), and CO_2_ emissions. The experimental samples totaled 47 sets of data from 1971 to 2017.  Figure 1 is a data view of each indicator. It can be seen that the five socioeconomic indicators related to CO_2_ emissions are showing an increasing trend year by year.

Table 1 presents the statistical analysis of the six variables. As shown in Table 1, the value of the standard deviation of CO_2_ emissions was found to be equal to 3.2443 × 10^6^. Further, the same statistics for FCC, PC, OC, GDP, and P were found to be equal to 5.302, 1.3254 × 10^3^, 610.2162, 2.3125 × 10^13^ and 1.6623 × 10^8^ respectively. The remainder of the results derived from this analysis is set out in Table 1.

### 3.2. Data Analysis

**Figure 2:**
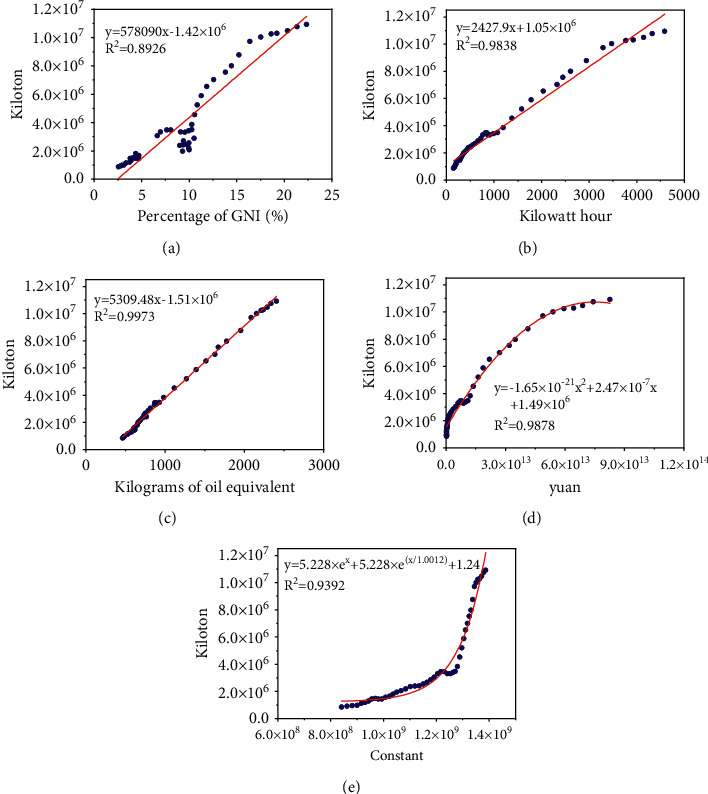
Cross-plots of economic indicators and CO_2_ emissions. (a) FCC-CO_2_ emissions. (b) PC-CO_2_ emissions. (c) OC-CO_2_ emissions. (d) GDP-CO_2_ emissions. (e) P-CO_2_ emissions.

In order to evaluate the relationship between socioeconomic-related indicators and CO_2_ emissions, this study used two analytical methods to characterize the sensitivity of various socioeconomic indicators to CO_2_ emissions. A single factor regression analysis was used to obtain a graph of the relationship between each indicator and CO_2_ emissions, as shown in Figure 2. In the regression analysis, the coefficient *R*^2^ is used to measure the degree of influence of different indicators on CO_2_ emissions, and *R*^2^ represents the proportion of the population variance, which can be obtained by

(13) $$\begin{array}{c} R^{2}=1-\frac{\sum_{i=1}^{n}\left(f\left(x_{i}\right)-y_{i}\right)}{\sum_{i=1}^{n}f{\left(x_{i}\right)}^{2}-\sum_{i=1}^{n}{\left(y_{i}\right)}^{2}/n}. \end{array}$$

It can be seen from Figure 2 that there is a positive correlation between each indicator and CO_2_ emissions, and *R*^2^ is very large, indicating that there is a good correlation between various economic indicators and CO_2_ emissions. Fixed capital consumption, electricity consumption, and oil consumption are all linearly related to CO_2_ emissions. However, both GDP and total population are nonlinearly related to CO_2_ emissions.

**Figure 3:**
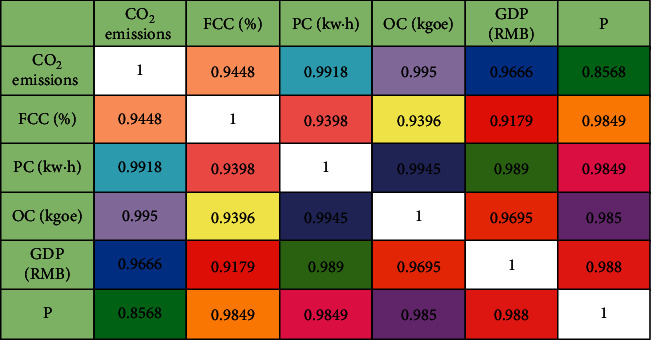
Graph of correlation matrix.

In addition to the simple regression method, we also used the correlation coefficient analysis method to analyze the relationship between economic indicators and CO_2_ emissions. The analysis results are shown in Figure 3. It can be seen that in addition to the total population indicators, other economic indicators have very high correlation coefficients. Both the crossplot method and the correlation coefficient analysis method show that there is a good correlation between the five socioeconomic indicators selected in this study and the CO_2_ emissions, which can be used as the input independent variable of the CO_2_ emission prediction model.

**Figure 4:**
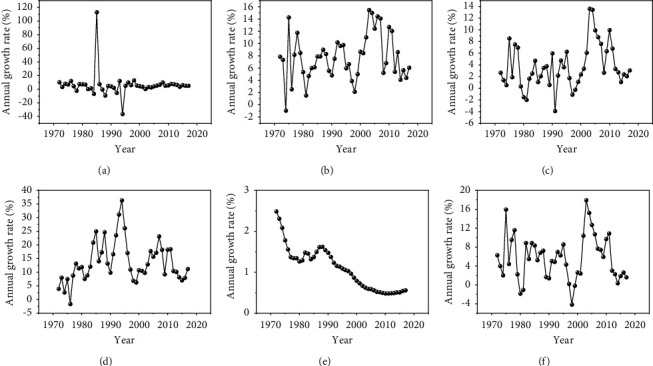
The annual growth rate of socioeconomic indicators and CO_2_ emissions. (a) FCC growth rates. (b) PC growth rates. (c) OC growth rates. (d) GDP growth rates. (e) P growth rates. (f) CO_2_ emissions growth rates.

In order to reflect the dynamic development of various social and economic indicators and the changes in CO_2_ emissions during the study period, we calculated the annual growth rates of various socioeconomic indicators and CO_2_ emissions.  Figure 4 shows the annual growth rate of socioeconomic indicators and CO_2_ emissions. It can be seen from the figure that except for a few years, the annual growth rate of FCC tends to be stable and the fluctuation is small; the annual growth rate of the population shows a trend of decreasing year by year. The annual growth rate of each parameter fluctuates greatly, and the change is relatively complicated.

**Figure 5:**
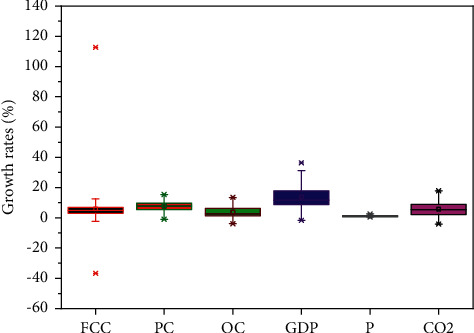
Boxplot showing the differences in values of each variable.

Table 2 presents the statistical analysis of growth rates for the six variables. As shown in Table 2, the value of the standard deviation of CO_2_ emissions growth rate was found to be equal to 4.7735. Further, the same statistics for FCC, PC, OC, GDP, and P growth rates were found to be equal to 17.7822, 3.7368, 3.7586, 7.4978, and 0.5206, respectively. The remainder of the results derived from this analysis is set out in Table 2.  Figure 5 presents the descriptive statistics in the form of a boxplot.

## 4. Results and Discussion

### 4.1. Model Establishment

**Figure 6:**
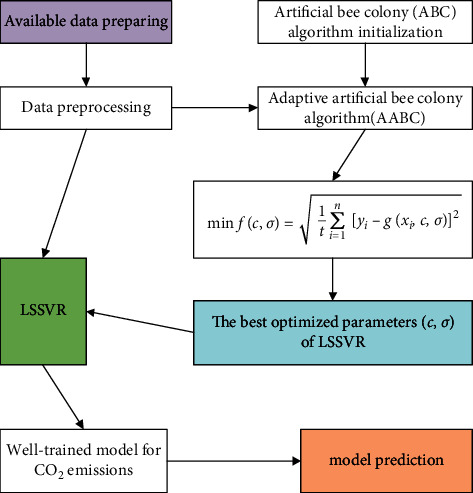
Flow chart of modeling for CO_2_ emissions prediction.

Formulas (7) and (9) in the original ABC algorithm are respectively replaced by the adaptive step size updated formula (11) and the adaptive honey source selection formula (12), composing an improved ABC algorithm called adaptive ABC algorithm. The AABC algorithm is combined with LSSVR to construct an artificial intelligence model for CO_2_ emissions prediction.  Figure 6 shows the flow of model construction. Considering that the dimensions of each socioeconomic indicator are different and the numerical values show large differences in magnitude, which is not conducive to modeling and calculation, we need to normalize the data first. Set the parameters of AABC, including the number of bee colonies *NP*, the maximum number of searches for honey sources limit, the maximum number of iterations *T* of the algorithm, the number of parameters to be optimized *D*, and the range of values of each parameter. Then, set the objective function. In this study, the minimum root mean square error (RMSE) fitted by LSSVR is taken as the objective function of the AABC algorithm, and its reciprocal is defined as the fitness function. Finally, through the iterative optimization of AABC algorithm, the optimal parameters of LSSVR are obtained; that is, the AABC-LSSVR model is successfully constructed, which can be used for the prediction of CO_2_ emissions.

### 4.2. Model Performance

As mentioned above, the data in this study are the socioeconomic and CO_2_ emissions data from 1971 to 2017 in China, totaling 47 groups. The 47 groups of data are divided into three parts: training, validation, and testing, of which 70% are trained. The validation and testing sections each accounted for 15%.

**Figure 7:**
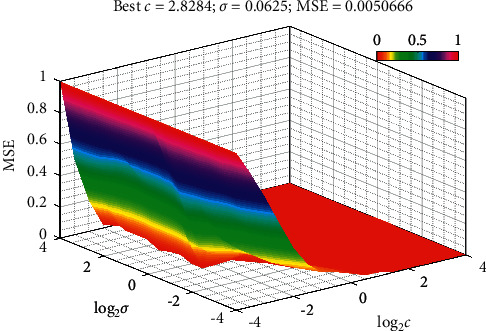
Parameter optimization results of LSSVR by using AABC algorithm.

When initializing the parameters of the ABC and AABC optimization algorithms, the size of the artificial bee colony is 50, the number of honey sources is half of the size of the bee colony, the dimension *D* of the solution problem is 2, the maximum number of iterations *T* is 300, and the maximum number of searches is limit 100. The penalty parameter *c* and the kernel function parameter *σ* have values ranging from [0.05, 1000] to [0.0001, 50], respectively. According to the aforementioned modeling process, the program was coded with MATLAB R2018b, which can be used to obtain the LSSVR and AABC-LSSVR models, respectively.  Figure 7 shows the optimization results of the AABC algorithm. The optimum *c* and *σ* were synchronously obtained as follows: best *c*=2.8284, best *σ*=0.0625.

**Figure 8:**
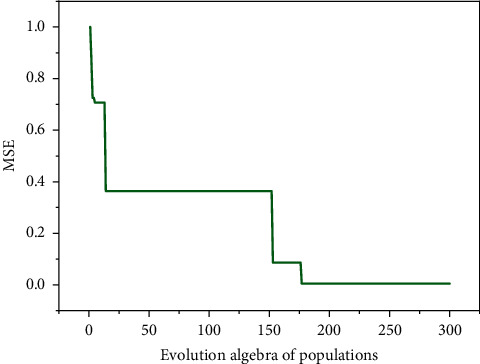
Iterative optimization curve of AABC-LSSVR model.

Figure 8 is an iterative optimization curve for the AABC-LSSVR model. It can be seen from the figure that under the control of adaptive regulation, in the early stage, the iterative curve drops rapidly (MSE value decreases rapidly), indicating that the iterative step size of the population is larger and the convergence speed is faster; in the medium term, the iterative curve is stable. The state indicates that some honey sources with low fitness are retained, so that the diversity of the population is supplemented; in the later stage, the iterative curve drops rapidly again to reach the convergence value, indicating that only the honey source with high fitness is selected at this time. It can be seen that the AABC has strong adaptability and effectiveness in searching for key parameters of the LSSVR algorithm.

**Figure 9:**
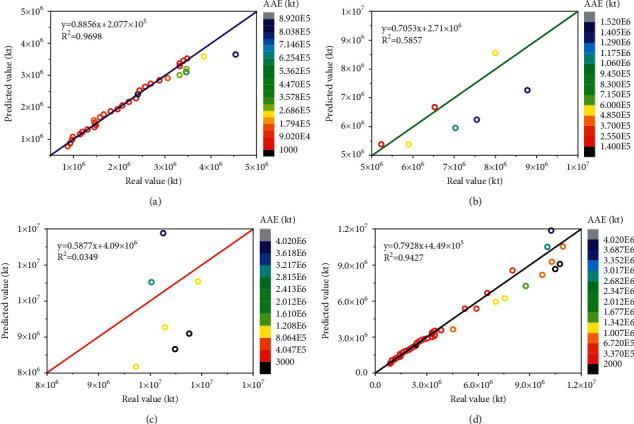
Comparison of the CO_2_ emissions prediction results with LSSVR model and real CO_2_ emissions. (a) LSSVR—training results. (b) LSSVR—validation results. (c) LSSVR—testing results. (d) LSSVR—all results.

**Figure 10:**
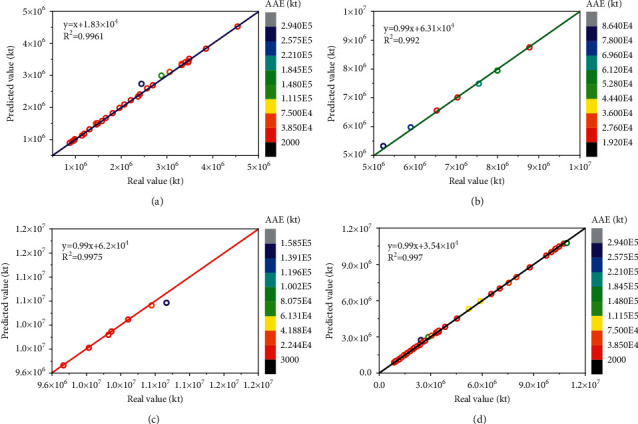
Comparison of the CO_2_ emissions prediction results with AABC-LSSVR model and real CO_2_ emissions. (a) AABC-LSSVR—training results. (b) AABC-LSSVR—validation results. (c) AABC-LSSVR—testing results. (d) AABC-LSSVR—all results.

The predictive performance of the LSSVR and AABC-LSSVR models with the same inputs is evaluated using the coefficient of determination (*R*^2^), as shown in Figures 9 and 10. It can be seen from Figures 9(a) and 10(a) that LSSVR has a strong fitting ability in the training set part, which is slightly inferior to the AABC-LSSVR model. However, as can be seen from Figures 9(b), 9(c) and 10(b), 10(c), the LSSVR model is significantly less effective in the verification and testing part than the AABC-LSSVR model, especially in the test section. It shows that the optimization effect of AABC on LSSVR is very obvious, which improves the fitting performance of LSSVR. It also shows that the AABC-LSSVR model proposed in this study has strong generalization ability in the prediction of CO_2_ emissions and is trustworthy.

Figures 9(d) and 10(d) show crossplot of real CO_2_ emissions versus predicted values. Coefficient of determination (*R*^2^) for LSSVR prediction is equal to 0.9427 which verifies robustness of LSSVR model. While coefficient of determination (*R*^2^) for AABC-LSSVR prediction is equal to 0.997 which verifies strong robustness of improved LSSVR model. It is sufficient to claim that the robustness of LSSVR was improved by using AABC algorithm.

**Figure 11:**
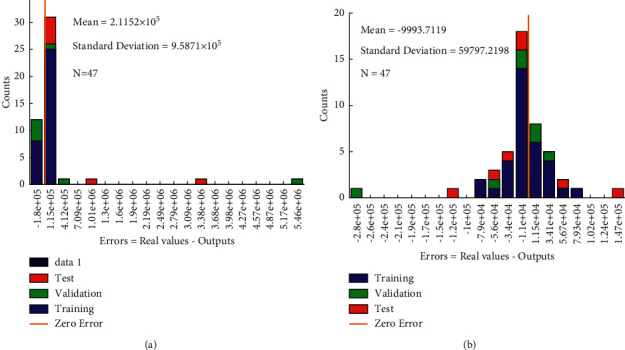
Absolute error histogram with 20 bins of each model. (a) LSSVR model. (b) AABC-LSSVR model.

In addition to coefficient of determination (*R*^2^), error distribution was also employed for assessment of the LSSVR and AABC-LSSVR performance.  Figure 11 allows more statistical analysis of LSSVR and AABC-LSSVR performance by using error distribution information. As shown in Figure 11(a), mean and standard deviation of error distribution for LSSVR model are in turn equal to 2.1152 × 10^5^ and 2.1152 × 10^5^, which are relatively larger than that of AABC-LSSVR model (see Figure 11(b)). It means that the prediction error of AABC-LSSVR model is more acceptable.

**Figure 12:**
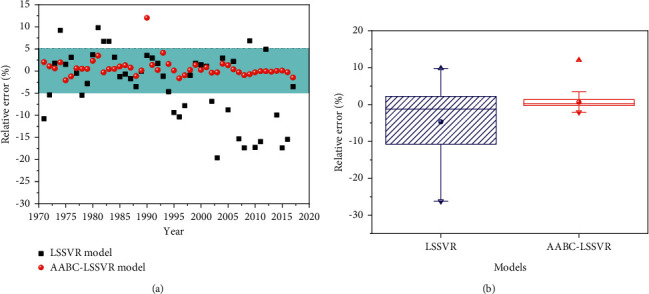
Results of relative error analysis for each model. (a) Relative error of models in prediction of CO_2_ emissions. (b) Boxplot showing the differences in relative error of each model.

Figure 12 provides an opportunity to compare relative error between LSSVR and AABC-LSSVR for all data points. From Figure 12(a), it can be seen that relative error of AABC-LSSVR for almost all data points is located in the range of [−5% 5%], which is an acceptable value.  Figure 12(b) presents the descriptive statistics in the form of a boxplot for relative error data. Small relative error of mean and fluctuation range of data reveals high performance of AABC-LSSVR model in CO_2_ emissions prediction.

In summary, we can see that AABC-LSSVR outperformed LSSVR owing to higher *R*^2^ and lower relative error. This study states AABC algorithm is very effective for LSSVR and AABC-LSSVR predicted values are in good agreement with reality.

### 4.3. Discussion

The model of CO_2_ emission prediction has been constructed based on AABC and LSSVR algorithms, and the performance of the model has been tested. The results also show that the AABC-LSSVR model has strong robustness and generalization ability, and the prediction accuracy is acceptable and trustworthy. Therefore, the AABC-LSSVR model will be used to predict China's CO_2_ emissions for the next five years (2018–2022). Considering that the socioeconomic indicators for the next five years are also unknown, this study will reduce the uncertainty of prediction by setting different scenarios, and these scenarios will adopt different input independent variable data. The characteristics of China's social and economic indicators have been analyzed in the past few years, and the annual growth rate of each indicator has been calculated. Therefore, four scenarios will be set to generate model input independent variable data, as shown in Table 3. These data are all calculated by (14). Scenario (a) is based on the growth rate calculated in 2017 as the compound annual growth rate. Scenario (b) is based on the average growth rate between 1971 and 2017 as the compound annual growth rate. Scenario (c) is based on the nonnegative minimum growth rate between 1971 and 2017 as the compound annual growth rate. Scenario (d) is based on the nonnegative maximum growth rate between 1971 and 2017 as the compound annual growth rate.

(14) $$\begin{array}{c} f\left(t_{n}\right)=f\left(t_{0}\right){\left(1+cagr\right)}^{n}, \end{array}$$

where *a* is the value after *n* years and *b* is the initial value.

**Figure 13:**
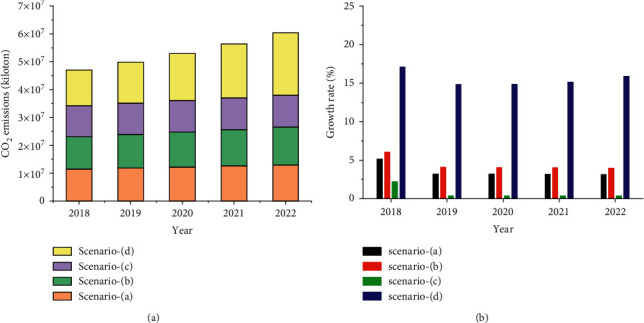
The forecast results of CO_2_ emissions for China in the next five years (2018–2022). (a) Prediction values of CO_2_ emissions in four scenarios. (b) The growth rates of CO_2_ emissions.

By substituting the five indicators of CO_2_ emissions in each of the scenarios in Table 3 into the AABC-LSSVR model, the predictions of CO_2_ emissions in four different scenarios can be obtained, as shown in Figure 13.

As can be seen from Figure 13(a), China's CO_2_ emissions in the next five years (2018–2022) have shown an upward trend year by year in the above four scenarios. In scenario (a), CO_2_ emissions increase from 1.0932 × 10^7^ kilotons in 2017 to 1.3026 × 10^7^ kilotons in 2022. In scenario (b), CO_2_ emissions increase from 1.0932 × 10^7^ kilotons in 2017 to 1.3575 × 10^7^ kilotons in 2022. The CO_2_ emissions in scenario (c) increase from 1.0932 × 10^7^ kilotons in 2017 to 1.1338 × 10^7^ kilotons in 2022. The CO_2_ emissions in scenario (d) increase from 1.0932 × 10^7^ kilotons in 2017 to 2.2509 × 10^7^ kilotons in 2022. It can be seen from Figure 13(b) that the annual growth rate of China's CO_2_ emissions in the next five years under the four scenarios is quite different. Scenario (a) has an average annual growth rate of 3.57%; scenario (b) has an average annual growth rate of 4.43%; scenario (c) has an average annual growth rate of 0.735%; scenario (d) has an average annual growth rate of 15.54%. In summary, the forecast of China's CO_2_ emissions in the next five years under the four scenarios indicates that China's CO_2_ emissions in 2022 will fluctuate between 1.1338 × 10^7^ kilotons and 2.2509 × 10^7^ kilotons.

The analysis results of the scenarios set above show that future CO_2_ emissions are closely related to their influencing factors, and changes in any key influencing factor will cause strong fluctuations in CO_2_ emissions. Referring to the social and economic development of China in the past period, prediction of CO_2_ emissions in the next few years using the hybrid intelligence model is proposed in this study. The results show that China's CO_2_ emissions will continue to rise in the future. The Chinese government should formulate special policies for relevant social-economic indicators to effectively control future CO_2_ emissions and complete the carbon peaking and carbon neutrality goals as soon as possible.

## 5. Conclusions

Based on China's CO_2_ emissions from 1971 to 2017 and related socioeconomic indicators, including FCC, electricity consumption, OC, GDP, and total population, a new hybrid intelligent algorithm model for China's CO_2_ emissions forecast is proposed in this research. In view of the shortcomings of the standard ABC algorithm, in this study, an adaptive step search formula and an adaptive probability selection formula are designed to enhance the optimization ability of ABC, thus forming the AABC algorithm to optimize the hyperparameters of the LSSVR model. Finally, the ABC-LSSVR and AABC-LSSVR models are established respectively to predict China's CO_2_ emissions. The results show that the absolute value of the relative error of the AABC-LSSVR model is almost controlled within 5%, which can confidently indicate that the AABC-LSSVR model has better accuracy and generalization ability for CO_2_ emissions prediction. It can be seen that the hyperparameter optimization problem of machine learning can be effectively solved by establishing a hybrid intelligent algorithm, thereby greatly improving the reliability of the prediction results in the research of CO_2_ emissions advance forecast. According to the forecast results of China's CO_2_ emissions from 2018 to 2022 by the AABC-LSSVR model, China's CO_2_ emissions will continue to grow in the future for a long time and will fluctuate between 1.1338 × 10^7^ kilotons and 2.2509 × 10^7^ kilotons by 2022. If the Chinese government needs to achieve the dual carbon goal as soon as possible, it is necessary to introduce targeted policies to restrict the social and economic indicators closely related to CO_2_ emissions, so as to effectively curb CO_2_ emissions in the future.

We make the following policy recommendations to the Chinese government based on the conclusions of the analysis above. First, in order to use fixed capital scientifically and rationally and reduce its physical loss as much as possible, it is necessary to improve the management awareness and management level of enterprises. Second, it is necessary to optimize the energy structure and increase the substitution ratio of new energy to traditional energy. China's energy structure is dominated by petrochemical energy, and a large amount of CO_2_ is released in the process of energy consumption. Therefore, increasing the proportion of clean energy such as solar energy and wind energy in the energy consumption structure can effectively reduce CO_2_ emissions, thereby alleviating the conflict between CO_2_ emissions and economic development. Third, China's industrial structure and export trade structure need to be adjusted. Manufacturing, which has long been China's main export sector, is highly dependent on upstream sectors, leading to more energy consumption and CO_2_ emissions. The modern service industry relies more on human capital investment, and its asset-light characteristics can greatly reduce the energy consumption of upstream and downstream industrial chains, which can reduce CO_2_ emissions from the source. Therefore, the transformation of the industrial structure from manufacturing to modern service industry can not only improve economic efficiency, but also reduce environmental costs. Fourth, China still needs to vigorously develop its economy to increase residents' income. There is a clear relationship between people's consumption concept and behavior and economic level; that is, the higher the social affluence is, the more likely people will pay attention to the environment and consider whether their behavior will have an impact on the environment. People in high-income societies will obviously reduce “high-carbon” consumption and are more inclined to buy low-carbon products to promote CO_2_ emission reduction. Fifth, according to the share of CO_2_ emissions, the government should focus on controlling high-emission sectors, such as electricity, heat, gas, and water production and supply industries, and encourage innovation and promotion of energy-saving technologies in these sectors. In addition, since technological progress is an effective way to reduce CO_2_ emissions, government departments should strongly support enterprises and scientific research institutions to develop low-carbon emissions, CO_2_ capture, and storage technology research and development. Sixth, China's urbanization is in an accelerated stage, and the government should vigorously promote a population structure that is conducive to sustainable development, to effectively control CO_2_ emissions. Human activities are the main reason for the increase of CO_2_ emissions. The government needs to guide people to green travel and low-carbon life, so that people can actively contribute to CO_2_ emission reduction through their own behaviors and achieve the purpose of effectively controlling the impact of population factors on CO_2_ emissions.

In addition, machine learning has become a key technology in CO_2_ emission forecasting research. Although the hybrid intelligence model proposed in this study has achieved good results, it still has the possibility of further optimization. CO_2_ emissions and related influencing factors are time series data. On the one hand, researchers can try to extract features from the original data to the maximum extent for algorithm modeling through feature engineering. On the other hand, researchers can try to expand the deep learning framework of the recurrent neural network series to this study. In short, only continuous in-depth research in the future can continuously improve the accuracy of advanced forecasting of CO_2_ emissions.

## Figures and Tables

**Table 1:** Summary of the social and economic indicators related to CO_2_ emissions for China.

	FCC (%)	PC (kw·h)	OC (kgoe)	GDP (RMB Yuan)	*P*	CO_2_ emissions (kiloton)
Minimum value	2.5739	151.9893	464.9332	2.4569 × 10^11^	8.4111 × 10^8^	8.7663 × 10^5^
Maximum value	22.3399	4.5916 × 10^3^	2.4066 × 10^3^	8.2712 × 10^13^	1.3864 × 10^9^	1.0932 × 10^7^
Average	9.6912	1.2906 × 10^3^	1.0732 × 10^3^	1.6128 × 10^13^	1.1599 × 10^9^	4.1852 × 10^6^
Standard deviation	5.3020	1.3254 × 10^3^	610.2162	2.3125 × 10^13^	1.6623 × 10^8^	3.2443 × 10^6^

**Table 2:** Summary of the social and economic indicators related to CO_2_ emissions for China.

	FCC	PC	OC	GDP	*P*	CO_2_ emissions
Initial growth rate	10.0202	7.8444	2.6317	3.887	2.4878	6.2675
Average growth rate	5.8882	7.7536	3.7044	13.7197	1.0936	5.7432
Minimum nonnegative growth rate	0.0872	1.4889	−3.9301	0.306	2.5942	0.4803
Maximum growth rate	112.7308	15.4519	13.5682	36.3418	2.4878	17.9247
Standard deviation	17.7822	3.7368	3.7586	7.4978	0.5206	4.7735

**Table 3:** The forecasting scenarios of the social and economic indicators related to CO_2_ emissions for China (from 2018 to 2022).

Scenario	Year	FCC (%)	PC (kw·h)	OC (kgoe)	GDP (yuan)	*P*
Scenario (a)	2018	24.5784	4.5917 × 10^3^	2.4699 × 10^3^	8.5927 × 10^13^	1.4209 × 10^9^
	2019	27.0412	5.3402 × 10^3^	2.5349 × 10^3^	8.9267 × 10^13^	1.4562 × 10^9^
	2020	29.7507	5.7591 × 10^3^	2.6017 × 10^3^	9.2737 × 10^13^	1.4925 × 10^9^
	2021	32.7318	6.2108 × 10^3^	2.6701 × 10^3^	9.6342 × 10^13^	1.5296 × 10^9^
	2022	36.0117	6.698 × 10^3^	2.7404 × 10^3^	1.0008 × 10^14^	1.5676 × 10^9^

Scenario (b)	2018	23.6613	4.9553 × 10^3^	2.4978 × 10^3^	9.4060 × 10^13^	1.4016 × 10^9^
	2019	25.0608	5.3478 × 10^3^	2.5925 × 10^3^	1.0697 × 10^14^	1.4169 × 10^9^
	2020	26.5432	5.7714 × 10^3^	2.6908 × 10^3^	1.2164 × 10^14^	1.4324 × 10^9^
	2021	28.1132	6.2286 × 10^3^	2.7928 × 10^3^	1.3833 × 10^14^	1.4481 × 10^9^
	2022	29.7762	6.7219 × 10^3^	2.8986 × 10^3^	1.5731 × 10^14^	1.4639 × 10^9^

Scenario (c)	2018	22.3593	4.6599 × 10^3^	2.4139 × 10^3^	8.4858 × 10^13^	1.3931 × 10^9^
	2019	22.3788	4.7293 × 10^3^	2.4214 × 10^3^	8.7059 × 10^13^	1.3997 × 10^9^
	2020	22.3983	4.7997 × 10^3^	2.4288 × 10^3^	8.9318 × 10^13^	1.4065 × 10^9^
	2021	22.4179	4.8712 × 10^3^	2.4362 × 10^3^	8.1635 × 10^13^	1.4132 × 10^9^
	2022	22.4375	4.9437 × 10^3^	2.4437 × 10^3^	8.4012 × 10^13^	1.4201 × 10^9^

Scenario (d)	2018	47.5238	5.301 × 10^3^	2.7332 × 10^3^	1.1277 × 10^14^	1.4209 × 10^9^
	2019	101.0978	6.1202 × 10^3^	3.1039 × 10^3^	1.5375 × 10^14^	1.4562 × 10^9^
	2020	215.0663	7.0658 × 10^3^	3.5251 × 10^3^	2.0963 × 10^14^	1.4925 × 10^9^
	2021	457.5122	8.1576 × 10^3^	4.0035 × 10^3^	2.8582 × 10^14^	1.5296 × 10^9^
	2022	973.2694	9.4181 × 10^3^	4.5466 × 10^3^	3.8968 × 10^14^	1.5676 × 10^9^

## Data Availability

The data used to support the findings of the study are available from the corresponding author upon request.
